# Performance of point-of-care international normalized ratio measurement to diagnose trauma-induced coagulopathy

**DOI:** 10.1186/s13049-017-0404-y

**Published:** 2017-06-21

**Authors:** Thomas Mistral, Yvonnick Boué, Jean-Luc Bosson, Pauline Manhes, Jules Greze, Julien Brun, Pierre Albaladejo, Jean-François Payen, Pierre Bouzat

**Affiliations:** 1 Grenoble Alpes Trauma Center, Pôle Anesthésie-Réanimation, CHU Grenoble Alpes, F-38000 Grenoble, France; 2 University Grenoble Alpes, F-38000 Grenoble, France; 3 INSERM U1216, F-38000 Grenoble, France; 4 Pôle Santé Publique, CHU Grenoble Alpes, F-38000 Grenoble, France

## Abstract

**Background:**

Trauma-induced coagulopathy (TIC) is a common feature after severe trauma. Detection of TIC is based upon classic coagulation tests including international normalized ratio (INR) value. Point-of-care (POC) devices have been developed to rapidly measure INR at the bedside on whole blood. The aim of the study was to test the precision of the Coagucheck® XS Pro device for INR measurement at hospital admission after severe trauma.

**Methods:**

We conducted a prospective observational study in a French level I trauma center. From January 2015 to May 2016, 98 patients with a suspicion of a post-traumatic acute hemorrhage had POC-INR measurement on whole blood concomitantly to classic laboratory INR determination (lab-INR) on plasma at hospital admission. The agreement between the two methods in sorting three predefined categories of INR (normal coagulation, moderate TIC and severe TIC) was evaluated using the Cohen’s kappa test with a quadratic weighting. The correlation between POC-INR and lab-INR was measured using the Pearson’s coefficient. We also performed a Bland and Altman analysis.

**Results:**

The agreement between the lab-INR and the POC-INR was moderate (Kappa = 0.45 [95% CI 0.36–0.50]) and the correlation between the two measurements was also weak (Pearson’s coefficient = 0.44 [95% CI 0.27–0.59]). Using a Bland and Altman analysis, the mean difference (bias) for INR was 0.22 [95% CI 0.02–0.42], and the standard deviation (precision) of the difference was 1.01.

**Discussion/conclusion:**

POC Coagucheck® XS Pro device is not reliable to measure bedside INR. Its moderate agreement with lab-INR weakens the usefulness of such device after severe trauma.

**Trial registration:**

NCT02869737. Registered 9 August 2016.

## Background

Trauma-induced coagulopathy (TIC) is a common phenomenon after severe trauma and is associated with transfusion requirements, risk of complications and mortality [1]. Early detection of TIC is based upon early and repeated monitoring of coagulation using a traditional laboratory determination of prothrombin time (PT), international normalized ratio (INR), activated partial thromboplastin time (APTT) platelet counts and fibrinogen [2]. Specifically, elevated INR was associated with death, multiple organ failure, and longer stay in hospital after severe trauma [3, 4]. This parameter also predicted accurately the requirement for red blood cell transfusion, including massive transfusion, in the context of trauma [5]. However, classic coagulation tests depend on laboratory processing, which are often delayed from the blood puncture. To overcome these limitations, point-of-care (POC) devices have been implemented to obtain a bedside assessment of the coagulation status on whole blood using viscoelastic method or bedside measurement of INR. Hence, POC-INR measurement device may provide a rapid, repeatable and low-cost measurement of real-time INR. Few studies evaluated POC devices for the diagnosis of TIC in the trauma bay [6–9]. In these studies, POC-INRs were obtained with significant gain in time compared with laboratory INRs (lab-INR), but their agreement with lab-INRs and their precision to diagnose TIC differed significantly. None of these studies used the Coagucheck® XS Pro device after severe trauma.

The aim of our study was to evaluate the precision of INR measurement with POC Coaguchek® XS Pro device at the admission of severe trauma patients. We hypothesized an almost perfect agreement between POC-INR measurement and lab-INR measurement.

## Methods

### Study design and patients

We conducted a prospective observational study in a level-I trauma center (Grenoble University Hospital, France) from January 2015 to May 2016. The Regional Institutional Ethics Committee (CECIC Rhône-Alpes-Auvergne, Clermont-Ferrand, IRB file number 2015–03, approval on January 13, 2015) approved the study design and, given its observational nature, waived the requirement for written informed consent. The study is recorded in clinicaltrials.gov, number: NCT02869737.

Inclusion criteria were patients older than 15 years-old admitted into the trauma bay for a suspicion of post-traumatic acute hemorrhage. Consecutive severe trauma patients were eligible if they had on admission at least one of the following items: 1) an hemodynamic instability defined by a systolic arterial blood pressure (SBP) ≤ 90 mmHg, 2) an hemodynamic stability (SBP > 90 mmHg) with the use of vasopressor or fluid therapy > 20 mL/kg, 3) a red blood cell transfusion before hospital admission, or 4) a specific post-traumatic lesion at high risk of coagulopathy: traumatic brain injury with a Glasgow Coma Score < 13 before any sedation, severe chest trauma with first recorded pre-hospital pulse oximetry ≤ 92%, abdominal injury with positive ultrasonography, penetrating trauma, suspicion of spine injury, suspicion of pelvic ring fracture, or proximal amputation. Non-inclusion criteria were pregnant women, or a medical history interfering with the coagulation process (severe hepatic failure, cholestasis, digestive malabsorption, oral anticoagulation, heparin-based anticoagulation treatment, or fat-soluble vitamin deficiency).

### Study protocol and data collection

Patients were prospectively included at hospital admission. The following clinical data were collected: age, sex, vital variables on admission (GCS, SBP, Heart rate, and SpO_2_), transfusion requirements, Injury Severity Score (ISS), Sequential organ failure assessment (SOFA) on day 1, length of stay in intensive care unit (ICU), and in-hospital mortality. Regarding biological data, blood samples were drawn for central laboratory analysis. Coagulation assays with citrated-tube were collected to measure prothrombin time (PT) for the calculation of lab-INR [INR = (PT patient / PT normal) ^ISI^, ISI: International sensitivity index], APTT, and fibrinogen concentration. The Lab-INR was performed on a STA-R evolution coagulometer (Stago, Asnières, France) using STA®-Neoplastine® CI Plus (Stago) reagent. The coefficient of variation for the lab-INR was between 2.9 and 4.9%. The average time from the blood puncture to the lab-INR measurement was approximately 30 min in our center. Concomitantly, an independent nurse measured POC INR from the same blood puncture (POC-INR) with the Coaguchek® XS Pro (Roche laboratory, Meylan, France) device. POC-INR values were recorded in a case report form (CRF), and were not transmitted to the physician in charge of the patient.

### POC measurements

All measurements were obtained with a unique device to maximize reproducibility. The POC Coaguchek® XS Pro consists of an amperometric determination of the PT after activation of the coagulation with human recombinant thromboplastin. Conditions of testing respected carefully constructor’s recommendations. At the patient’s bedside, whole blood from venous puncture was used to release a drop (at least 8 μl) on a specific strip, which was inserted into the hand-sized device to provide POC-INR. ISI of Coaguchek® XS Pro was 1.0. The procedure was easy-going for any qualified nurse, and very fast (approximately 1 min). Two types of quality control were performed: each strip was tested before use to detect deteriorated strips and the POC device was itself monitored monthly with a specific control-kit as recommended by the constructor. These tests were mandatory to perform POC-INR measurements and could not be overdriven. The coefficient of variation for the POC-INR was measured between 3.4 and 6% by the company.

### Endpoints

Primary outcome was the concordance in categorical sorting between POC-INR and lab-INR on admission. INR values were sorted in three categories: 1) normal INR value: INR < 1.2; 2) moderate TIC: 1.2 ≤ INR <1.5; 3) severe TIC: INR ≥ 1.5 according to Frith et al. [3] and Peltan at al. [4].

Secondary outcomes were 1) the correlation between POC-INR and lab-INR values, and 2) the characteristics of the trauma population according to their lab-INR category.

### Study size

To be clinically relevant, we expected a kappa coefficient equal to 0.95 between POC-INR and lab-INR. The number of patients to include was set at 100 to obtain a 95% confidence interval (95% CI) between 0.85 and 0.99.

### Statistical analysis

Descriptive statistics included frequencies for categorical variables, and median values (25th–75th percentiles) for continuous variables. The concordance between POC-INR and lab-INR in sorting the three pre-defined classes of INR was analyzed using Cohen’s Kappa test with a quadratic weighting. Using the laboratory measurement as the gold standard, we performed a linear regression analysis to test the correlation between lab-INR and POC-INR and calculated the Pearson correlation coefficient. Statistical analysis was performed with the software STATA 13.1 (Stata Corp®, College Station, TX). Pearson’s coefficients and Kappa test were presented with their 95% confidence interval (95% CI). Agreement between the two methods was also assessed by the method of Bland and Altman, calculating the mean difference (bias, d) with the standard deviation of the differences (precision, s) and the limits of agreement (d ± 2 s).

## Results

**Fig. 1:**
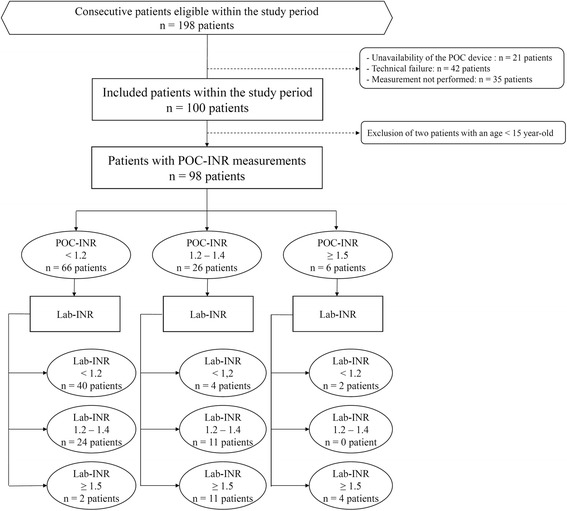
Flow chart of the study population

**Fig. 2:**
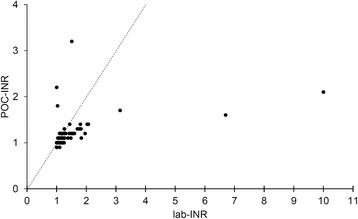
Scatter plot of point-of-care INR values (POC-INR, Y-axis) against laboratory INR measurements (lab-INR, X-axis) for the 98 patients. The correlation between these values was weak with a Pearson’s coefficient equal to 0.44 [95% CI 0.27–0.59]. The *dash-line* represents the ideal linear relationship between the two methods

**Fig. 3:**
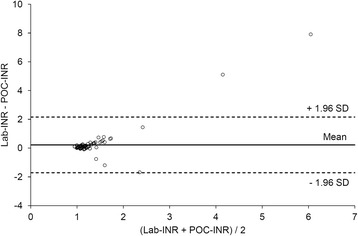
Bland and Altman plot. The difference between the laboratory INR (lab-INR) and the point-of-care INR (POC-INR) is plotted against the mean of lab-INR and POC-INR for 98 paired measurements in the study. For each data point, the mean value [(lab-INR + POC-INR)/2] is on the x axis and the difference (lab-INR – POC-INR) on the y axis

Flow chart of eligible patients and reasons for non-inclusion are presented in Fig. 1. Within the study period, 100 patients were included. As two patients with an age lower than 15-year-old were wrongly included, 98 severe trauma patients were considered for final analysis with no missing data on the primary outcome (complete case analysis). Patient’s characteristics are presented in Table 1. Median lab-INR was 1.2 [1.1–1.3], with 46 patients with normal coagulation state, 35 patients with moderate TIC, and 17 patients with severe TIC. Characteristics of the study population according to their lab-INR category are presented in Table 1. The agreement between the POC-INR the lab-INR in sorting the three predefined classes of INR was moderate with a kappa equal to 0.45 [95% CI 0.36–0.50] (Table 2). The correlation between POC-INR values and lab-INR values was weak with a Pearson’s coefficient equal to 0.44 [95% CI 0.27–0.59] (Fig. 2). Using Bland-Altman analysis, the overall mean difference (bias, d) was 0.22 [95% CI 0.02–0.42], and standard deviation of the differences (precision, s) was 1.01. Figure 3 presents the limits of agreement (d ± 1.96 s, from −1.76 to 2.20).

**Table 1:** Characteristics of the whole study population and according to their coagulation status on admission. Normal coagulation status was defined by a laboratory INR (lab-INR) < 1.2, moderate trauma-induced coagulopathy (TIC) by 1.2 ≤ lab-INR <1.5, and severe TIC by a lab-INR ≥ 1.5

Variables	Lab-INR < 1.2 *n* = 46 patients	Lab-INR [1.2–1.5] *n* = 35 patients	Lab-INR ≥ 1.5 *n* = 17 patients	Total population *N* = 98 patients
Age, years	42 [30–55]	41 [22–55]	44 [35–48]	42 [27–55]
Male, n (%)	41(89)	34 (97)	17 (100)	92
Blunt trauma, n (%)	40 (87)	33 (94)	13 (76)	86
Patients with pre-hospital fluid therapy > 20 ml/kg, n (%)	24 (52)	18 (51)	11 (65)	53
Patients with pre-hospital RBC transfusion, n (%)	1 (2)	2 (6)	3 (18)	6
Patients with pre-hospital mechanical ventilation, n (%)	10 (22)	17 (49)	11 (65)	38
First recorded pre-hospital pulse oximetry (SpO2), %	96 [92–98]	94 [87–97]	48 [0–96]	96 [89–98]
Patients with first recorded SpO2 ≤ 92%, n (%)	9 (20)	10 (29)	3 (18)	22
Vital variables on admission				
Heart rate, Beats/min	80 [70–105]	93 [70–110]	95 [75–100]	89 [10–105]
Systolic arterial blood pressure, mmHg	120 [110–140]	120 [110–135]	90 [75–110]	120 [103–140]
SBP ≤ 90 mmHg, n (%)	4 (9)	4 (11)	11 (65)	19
Glasgow Coma Scale before sedation	15 [12–15]	11 [6–15]	3 [3–14]	14 [6–15]
Patients with GCS < 13, n (%)	12 (26)	18 (51)	10 (59)	40
Patients treated with tranexamic acid, n (%)	12	8	12	32
Patients with vasopressor on admission, n (%)	20 (43)	18 (51)	16 (94)	54
Positive Focused Assessment Sonography for Trauma, n (%)	12 (26)	15 (43)	10 (59)	37
POC INR on admission	1.0 [1.0–1.1]	1.1 [1.1–1.2]	1.3 [1.2–1.4]	1.1 [1.0–1.2]
Laboratory coagulation variables on admission				
Prothrombin time (PT), %	92 [87–100]	72 [70–77]	45 [40–53]	79 [70–92]
INR	1.1 [1.0–1.1]	1.2 [1.2–1.3]	1.8 [1.6–2.0]	1.2 [1.1–1.3]
Activated partial thromboplastin time (APTT), sec	28.8 [27.2–31.4]	30.8 [29.8–33.7]	50.5 [37.1–57.5]	30.8 [28.6–35.4]
Fibrinogen concentration, g/L	2.7 [2.4–3.0]	2.2 [2.0–2.6]	1.4 [1.0–1.6]	2.3 [2–2.8]
Serum lactate concentration on admission, g/L	1.7 [1.0–2.7]	2.0 [1.3–3.7]	5.7 [3.5–9.1]	2.2 [1.4–4.3]
Patients with RBC transfusion within 24 h, n (%)	3 (7)	5 (14)	12 (71)	20
Patients with FFP transfusion within 24 h, n (%)	1 (2)	3 (9)	11 (65)	15
Injury Severity Score (ISS)	25 [14–29]	25 [13–38]	34 [25–43]	25 [16–34]
Sequential organ failure assessment (SOFA) at day 1	3 [0–5]	5 [0–7]	8 [5–10]	4 [0–7]
Length of stay in ICU. days	5 [2–10]	8 [3–14]	1 [1–10]	5 [1–12]
In-hospital mortality. n (%)	3 (7)	5 (14)	8 (47)	16

Data are median (25th-75th percentiles). *FFP* fresh frozen plasma, *ICU* intensive care unit, *ISS* Injury Severity Score, *RBC* red blood cell

**Table 2:** Concordance between the laboratory INR (lab-INR) and the point-of-care INR (POC-INR) in sorting three INR categories: normal INR (INR <1.2). moderate trauma-induced coagulopathy (TIC) (1.2 ≤ INR <1.5). and severe TIC (INR ≥1.5). Bolded values represent the number of patients accordingly classified by both methods

	lab-INR <1.2	1.2 ≤ lab-INR <1.5	lab-INR ≥1.5	Total
POC-INR <1.2	**40**	24	2	66
1.2 ≤ POC-INR <1.5	4	**11**	11	26
POC-INR ≥1.5	2	0	**4**	6
Total	46	35	17	98

Values are numbers. *INR* international normalized ratio, *lab* laboratory, *POC* point-of-care

Bold values are correct concordance between the two methods

## Discussion

Early diagnosis of TIC at hospital admission is part of the global medical strategy for the management of severe trauma patients. Using a POC-INR measurement device at the bedside, we found a moderate agreement between this POC method and the classic laboratory determination of INR. The correlation between these two methods was not acceptable and the precision of POC-INR was poor. Taken together, these findings challenged the usefulness of this POC-INR measurement device to diagnose TIC at the bedside.

POC-INR devices were originally implemented to monitor patients with oral anticoagulation by vitamin-K antagonist [10–15]. Potential utilization have been extended to monitor coagulation state in the operating room [16], in the military setting [17], in emergency departments [18], and in the pre-hospital field [19]. From the severe trauma standpoint, the main interest of a bedside INR assessment lies in early diagnosis of trauma-induced coagulopathy. Bedside and real-time measurement of the INR would allow physicians to individualize hemostatic treatment while avoiding futile transfusion of coagulation factors. Despite encouraging preliminary reports, our study showed poor precision of POC-INR to estimate lab-INR. Indeed, its agreement and its correlation with lab-INR were not sufficient for clinical use. For instance, the limits of agreement between the two methods using Bland and Altman analysis were −1.96 (lower limit of agreement) and + 2.20 (upper limit of agreement). Considering that INR is mostly measured between 1 and 3, such differences are relevant from the clinical standpoint. Specifically, the Fig. 2 revealed possible high discrepancy between the POC method and the lab method in two patients (lab-INR equal to 6 and 10, whereas POC-INR was lower than 2). These discrepancies were repeatedly controlled in these two patients and were not related to either lab-INR errors or blood contamination. Our results are inconsistent with a previously reported correlation between lab-INR and POC-INR using the INRatio Monitoring-System (Hemosense, Milpitas, CA) in severe trauma patients but are consistent with results obtained with the Hemochron Signature Elite device (International Techidyne Corporation, Edison, NJ) in the context of acute hemorrhage and with another POC-INR device after severe trauma [8]. To our knowledge, our study is the largest prospective cohort of severe trauma patients that evaluates a POC-INR device at hospital admission. Methodological factors could explain the disagreement between POC-INR measurements and laboratory values. The POC device uses whole blood and is calibrated for normal hematocrit and platelet counts, whereas lab-INRs are measured on plasma. After severe trauma, fluctuations of hematocrit or platelet counts are susceptible to interfere with POC measurements. In the laboratory, the use of platelet-depleted plasma through centrifugation decreases inter-individual differences induced by changes in platelet count. These considerations on pre-analytical technique may account for moderate agreement between the two methods. Another explanation may be the influence of fluid resuscitation on blood composition after severe trauma. The use of crystalloids or macromolecules for fluid loading contributes to the modification of blood in resuscitated patients and may affect the precision of the POC method. In our study, a high proportion of patients had fluid therapy > 20 ml/kg, which probably accounted for the disagreement between the two methods. According to French guidelines, patients were also largely treated with vasopressor. The influence of vasopressor use on POC-INR measurements remains unknown but might be limited as compared to the effect of fluid loading. Finally, changes in fibrinogen concentration are also critical to interpret the disagreement between the laboratory method and the POC method. The POC measurement of INR is based upon an electrochemical method using a thrombin substrate and, thus, is not sensitive to fibrinogen concentration. Conversely, the lab-INR determination is based upon a mechanical detection of the clot formation, which is dependent on fibrinogen concentration. As a result, variation in fibrinogen concentration largely affected the concordance between the two methods.

We acknowledge several limits of our study. First, few patients presented with severe acute traumatic coagulopathy and the concordance between the POC device and the laboratory would be of interest in a more diverse trauma population. Second, a low proportion of eligible patients were finally analyzed and only 98 patients out of 100 included patients were considered after the exclusion of two wrongly included patients. Reasons for exclusion are detailed in Fig. 1 and our cohort could not be considered as consecutive. However, we experienced technical failure with the POC device and its frequent calibration limits the availability of the device 24/7. Third, the definition of TIC was based upon INR measurements, which might not reflect all aspects of coagulation impairment related to trauma, like platelet dysfunction or fibrinolysis. Although our study disqualifies the use of POC-INR to assess coagulation process after severe trauma, we think that viscoelastic methods are more comprehensive and we encourage the development of such bedside assessment in severe trauma.

## Conclusion

POC INR device was not sufficiently reliable to promote its use in the trauma bay for bedside INR measurement. Poor agreement between this technique and the classic laboratory test may compromise the diffusion of such device for the management of severe trauma patients.
